# Why Do HIV-1 and HIV-2 Use Different Pathways to Develop AZT Resistance?

**DOI:** 10.1371/journal.ppat.0020010

**Published:** 2006-02-17

**Authors:** Paul L Boyer, Stefan G Sarafianos, Patrick K Clark, Eddy Arnold, Stephen H Hughes

**Affiliations:** 1 HIV Drug Resistance Program, NCI-Frederick, Frederick, Maryland, United States of America; 2 Center for Advanced Biotechnology and Medicine and Department of Chemistry and Chemical Biology, Rutgers University, Piscataway, New Jersey, United States of America; 3 Basic Research Program, SAIC-Frederick, Inc., NCI-Frederick, Frederick, Maryland, United States of America; University of Pennsylvania School of Medicine, United States of America

## Abstract

The human immunodeficiency virus type 1 (HIV-1) develops resistance to all available drugs, including the nucleoside analog reverse transcriptase inhibitors (NRTIs) such as AZT. ATP-mediated excision underlies the most common form of HIV-1 resistance to AZT. However, clinical data suggest that when HIV-2 is challenged with AZT, it usually accumulates resistance mutations that cause AZT resistance by reduced incorporation of AZTTP rather than selective excision of AZTMP. We compared the properties of HIV-1 and HIV-2 reverse transcriptase (RT) in vitro. Although both RTs have similar levels of polymerase activity, HIV-1 RT more readily incorporates, and is more susceptible to, inhibition by AZTTP than is HIV-2 RT. Differences in the region around the polymerase active site could explain why HIV-2 RT incorporates AZTTP less efficiently than HIV-1 RT. HIV-1 RT is markedly more efficient at carrying out the excision reaction with ATP as the pyrophosphate donor than is HIV-2 RT. This suggests that HIV-1 RT has a better nascent ATP binding site than HIV-2 RT, making it easier for HIV-1 RT to develop a more effective ATP binding site by mutation. A comparison of HIV-1 and HIV-2 RT shows that there are numerous differences in the putative ATP binding sites that could explain why HIV-1 RT binds ATP more effectively. HIV-1 RT incorporates AZTTP more efficiently than does HIV-2 RT. However, HIV-1 RT is more efficient at ATP-mediated excision of AZTMP than is HIV-2 RT. Mutations in HIV-1 RT conferring AZT resistance tend to increase the efficiency of the ATP-mediated excision pathway, while mutations in HIV-2 RT conferring AZT resistance tend to increase the level of AZTTP exclusion from the polymerase active site. Thus, each RT usually chooses the pathway best suited to extend the properties of the respective wild-type enzymes.

## Synopsis

Although a number of useful drugs have been developed to treat HIV-1 infections, the virus can become resistant to all of the drugs. Resistance involves the acquisition of mutations in the part of the virus that is the drug target. AZT resistance involves mutations in the viral enzyme reverse transcriptase (RT); RT copies the viral genetic information from RNA into DNA. HIV-1 can develop AZT resistance in two ways; each resistance pathway is characterized by distinct RT mutations. One of the pathways is used by the vast majority of AZT-resistant viruses isolated from patients. However, the closely related virus HIV-2 appears to use the other pathway more frequently than HIV-1. The authors compared the structures and biochemical properties of HIV-1 and HIV-2 RT to try to understand why these two related viruses acquire different AZT-resistance mutations. Although the two RTs are biochemically and structurally similar, there are differences in the respective wild-type RTs, and each can be considered to be part way along the two different resistance pathways. For this reason, it is easier for each virus to acquire the mutations that allow it to use the resistance pathway that extends the properties of the respective wild-type RTs.

## Introduction

Although considerable progress has been made in developing successful anti-human immunodeficiency virus type 1 (HIV-1) drugs and drug therapies, there are serious problems with drug toxicity and the development of drug-resistant viral strains. HIV-1 can develop resistance to all 21 of the currently approved drugs to treat it. Of these 21 drugs, 13 inhibit the virally encoded reverse transcriptase (RT). The available RT inhibitors can be divided into two classes: nucleoside reverse transcriptase inhibitors (NRTIs) and nonnucleoside inhibitors (NNRTIs). Both classes of inhibitors block the polymerase activity of RT. NNRTIs bind in a hydrophobic pocket in HIV-1 RT near the polymerase active site. A bound NNRTI does not block the binding of either the nucleic acid substrate or the incoming 3′-deoxynucleotide triphosphate (dNTP); however, it does block the chemical step of polymerization. NRTIs are analogs of the normal nucleotides used to synthesize viral DNA; however, NRTIs lack a normal 3′-OH, and, as a consequence, act as chain terminators when incorporated into viral DNA by RT. Most NRTIs are given to patients in an unphosphorylated state (the exception is tenofovir, a nucleotide analog that is given as a pro-drug). NRTIs must be taken up by cells and phosphorylated by cellular enzymes before they can be incorporated by RT [1] and references in [1].

Resistance to NRTIs is caused by mutations in HIV-1 RT. Resistance implies that the mutant RT has an enhanced ability to discriminate between the NRTI and normal nucleosides compared to wild-type RT. There are two different ways in which this increased discrimination can occur: 1) the mutant RT incorporates the NRTI triphosphate (NRTITP) less efficiently than does the wild-type RT; 2) the mutant RT is more efficient at selectively excising the nucleoside analog reverse transcriptase inhibitor triphosphate (NRTIMP) after it has been incorporated into viral DNA than is wild-type RT. Both mechanisms are used by NRTI-resistant HIV-1 mutants. For example, the M184V mutation selectively interferes with the incorporation of 3TCTP by steric hindrance [1]. In contrast, the most common form of AZT resistance (which usually involves some combination of the mutations M41L, D67N, K70R, T215F/Y, and K219E/Q) causes the selective excision of AZTMP [1–4].

We have developed a model that explains the mechanism that underlies the selective excision of AZTMP by AZT-resistant HIV-1 RT [3]. HIV-1 RT does not have a separate nuclease activity that carries out AZTMP excision; the excision reaction is closely related to the polymerase reaction run in reverse. If the polymerase reaction is run in reverse, the products of the polymerization reaction (the elongated DNA primer and pyrophosphate) are brought back together at the polymerase active site, and the last nucleotide at the 3′ end of the primer is joined to pyrophosphate, regenerating a dNTP and shortening the primer by one nucleotide. In the excision reaction that causes AZT resistance in vivo, the pyrophosphate donor is ATP, and the reaction that removes the last nucleotide from the primer generates a dinucleoside tetraphosphate [1,3,4]. The model resolves two important questions: 1) How do the mutations in HIV-1 RT cause AZT resistance? 2) Why is excision much more efficient for AZT than for other NRTIs? The canonical AZT-resistance mutations cause AZT resistance because they enhance the ability of HIV-1 RT to bind ATP appropriately, thus enhancing the excision reaction. The reason AZTMP is excised much more efficiently than other NRTIMPs is that it has better access to the nucleotide binding site (N site), where excision can occur [3]. However, the virus can acquire additional mutations that enhance its ability to excise a broader range of NRTIs. The most common of these are insertions in the fingers subdomain (usually an insertion of two amino acids between positions 69 and 70, accompanied by mutations at what were originally amino acids 69 and 70). The fingers insertion mutations appear to give NRTIMP-terminated primers better access to the N site, where the NRTIMP can be excised [5–7].

The excision mechanism accounts for the vast majority of HIV-1 AZT resistance; however, in some cases, particularly in cases in which patients were treated with AZT in combination with ddI and ddC, HIV-1 develops resistance via the Q151M mutation. This mutation has been observed in only 5% of the patients treated with NRTIs, but the Q151M mutation, by itself, appears to have only a slight deleterious effect on the polymerase activity of RT [1]. The Q151M mutation interferes with the incorporation of AZTTP rather than enhancing the excision of AZTMP [1,8–10].

Although far fewer HIV-2–infected patients have been treated with AZT, it appears that the predominant pathway for AZT resistance in HIV-2 involves mutations at Q151 (usually Q151M; however Q151I and Q151L mutations have been reported) rather than the excision pathway, and it has been suggested that the preferred pathways for AZT resistance may be different for the two viruses [11–16]. We have used biochemical analysis and structural comparisons to try to understand why these two related viruses prefer to use different AZT resistance mechanisms. In simple polymerase assays, HIV-1 RT and HIV-2 RT have similar levels of polymerase activity. Wild-type HIV-1 RT readily incorporates AZTTP, but it also removes AZTMP from the end of a blocked primer relatively efficiently using an ATP-mediated excision reaction [1–4]. In contrast, wild-type HIV-2 RT incorporates AZTTP less efficiently; but is also less efficient in removing AZTMP in an ATP-mediated excision reaction. This suggests that HIV-1 RT has a better nascent ATP binding site than HIV-2 RT. A comparison of the structures of HIV-1 RT and HIV-2 RT shows that there are a number of significant differences in the region that forms the putative ATP binding site [3] that could account for the difference in the ability of the two enzymes to bind ATP for the excision reaction. As mentioned above, HIV-2 RT incorporates AZTTP less efficiently than HIV-1 RT. We compared the regions around the polymerase active site and dNTP binding site (with a particular focus on Q151) in HIV-1 RT and HIV-2 RT to try to account for this difference. There are modest differences in this region that suggest that there are differences in the details of how the two RTs interact with an incoming dNTP. Some of these differences could contribute to the preference HIV-2 RT shows for choosing the Q151M pathway for AZT resistance.

## Results

### Comparing HIV-1 RT and HIV-2 RT

There are published data to suggest that HIV-1 RT and HIV-2 RT have similar levels of polymerase activity [17,18]. To confirm this, we measured the polymerase activities of HIV-1 RT and HIV-2 RT. We found that the two enzymes have similar activities in standard assays; it appears that HIV-2 RT is slightly less active in a simple polymerase assay (~75% of HIV-1 RT) and slightly less processive than HIV-1 RT on a single-stranded M13 DNA template (Figure 1). We also compared the RNase H activities of the two enzymes; as expected from the published data, HIV-1 RT has a more active RNase H than HIV-2 RT (unpublished data). We measured the ability of HIV-1 RT to incorporate AZTTP in a simple polymerase assay: HIV-1 RT is significantly more susceptible to inhibition by AZTTP than is HIV-2 RT (Figure 2). This is in agreement with a recent study suggesting that HIV-1 viral replication is more sensitive to AZT than is HIV-2 [16]. We also measured the kinetics of incorporation of dTTP and AZTTP in a single nucleotide incorporation assay (Table 1). HIV-1 RT had a higher k_cat_ and a lower K_m_ for both dTTP and AZTTP compared with HIV-2 RT (Table 1). Because AZT is poorly phosphorylated by cellular kinases, the differences in the K_m_ for AZTTP may be particularly important and may account for the difference in susceptibility of the two enzymes to inhibition by AZTTP (see Discussion).

**Figure 1:**
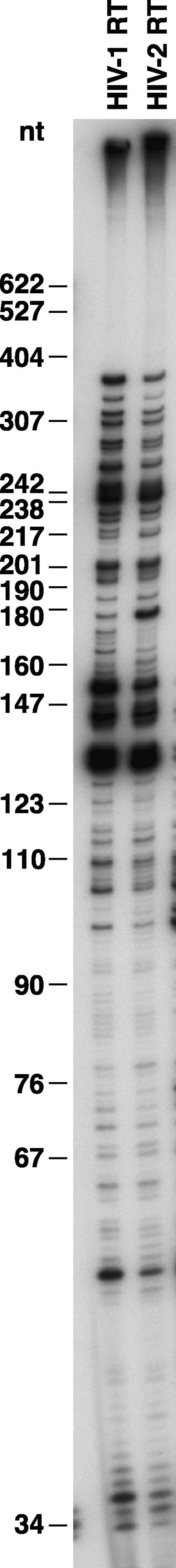
Processivity of Wild-Type HIV-1 and HIV-2 RTs As described in Materials and Methods, a 5′ end-labeled primer was annealed to single-strand M13mp18 DNA, then extended with wild-type HIV-1 RT or HIV-2 RT in the presence of 10.0 μM of each dNTP and unlabeled poly(rC)•oligo(dG), which acts as a “cold trap.” The cold trap limits extension to one round of polymerization. The location of the size marker bands (in nucleotides) are shown on the left.

**Figure 2:**
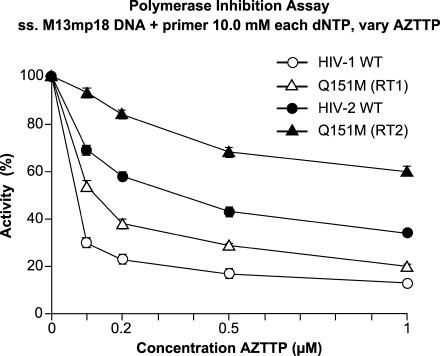
Inhibition of RT Polymerization by AZTTP The assay was done as previously described [1]. Normal dNTPs were present in the reaction at 10.0 μM each. Increasing concentrations of AZTTP were added, and the level of radioactive [α−^32^P]dCTP incorporated into the template/primer was measured. The level of radioactivity incorporated in the absence of analog was considered 100% activity; the other reactions were normalized to this value. The error bars are included; however, they are partially obscured by the data symbols. RT1 and RT2 designate the HIV RT backbone in which the Q151M mutation was made: HIV-1 or HIV-2.

**Table 1:**
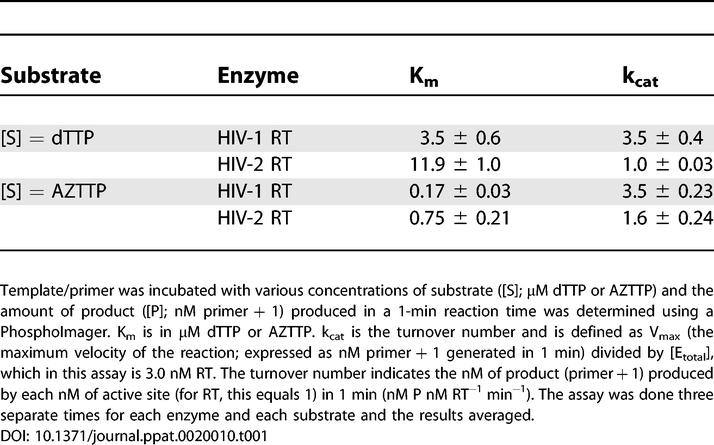
Summary of the Single Nucleotide Incorporation Kinetics Assay

We examined the effects of the Q151M mutation on both HIV-1 RT and HIV-2 RT activities. Both wild-type HIV-1 RT and the HIV-1 RT containing Q151M had similar polymerase activity when M13mp18 DNA was used as the template (the Q151M variant had 105% of the activity of wild type HIV-1 RT). As expected, the Q151M mutation decreased the sensitivity of HIV-1 RT to AZTTP (Figure 2). In HIV-2 RT, the Q151M mutation had a small effect on polymerase activity (93% of the activity of the wild-type HIV-2 RT) in the M13mp18 polymerase assay. However, the Q151M mutation made HIV-2 RT relatively resistant to AZTTP (Figure 2).

We also ran the polymerase reaction in reverse (as part of an excision/extension assay with pyrophosphate as the pyrophosphate donor). HIV-1 RT is slightly more active in a simple polymerization assay; however, it also incorporates AZTTP more readily than HIV-2 RT, and it might be expected to excise AZTMP more readily in a pyrophosphate-based excision assay. Because the excision/extension assay involves both incorporation and excision, these factors are likely to balance out. As Figure 3A shows, HIV-1 RT and HIV-2 RT behave similarly when pyrophosphate is used in an excision/extension assay. However, when ATP was used as the pyrophosphate donor in an excision/extension assay, HIV-1 RT was markedly more efficient at removing AZTMP from the end of a blocked primer (Figure 3B). Given the fact that the two enzymes showed equivalent levels of AZTMP excision in a similar assay with pyrophosphate as the donor, this result suggests that HIV-1 RT has a better nascent ATP binding site than HIV-2 RT. To test this hypothesis, we compared the structure of the putative ATP binding site of HIV-1 RT with the equivalent region of HIV-2 RT using the recently published structure of HIV-2 RT [19,20].

**Figure 3:**
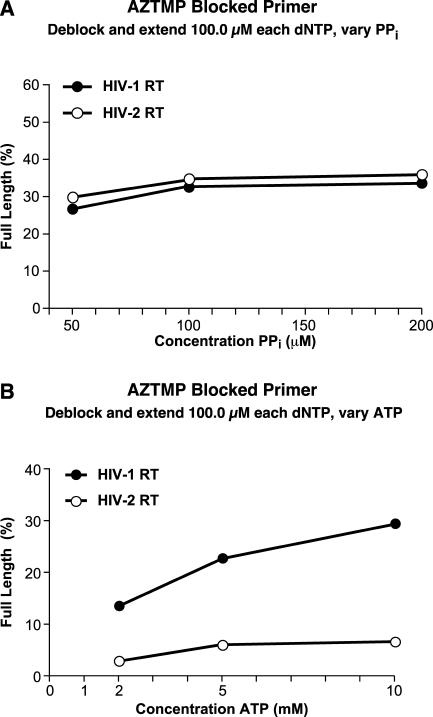
Excision/Extension of AZTMP by HIV-1 RT and HIV-2 RT As described in Materials and Methods, a 5′ end-labeled primer was annealed to a template. The 3′ end of the primer was blocked by the addition of AZTMP. (A) The excision/extension assays were performed with AZTMP-blocked primers in the presence of high levels of dNTPs (100.0 μM each), which favors formation of the ternary complex (RT/DNA/dNTP) and the indicated concentrations of NaPPi (50.0, 100.0, 200.0 μM). All experiments were done at least in duplicate; a typical result is shown. (B) Excision/extension of an AZTMP-blocked primer using high levels of dNTPs (100.0 μM each dNTP) and the indicated concentrations of ATP (2.0, 5.0, 10.0 mM). All experiments were done at least in duplicate; a typical result is shown.

### Comparing the Putative ATP Binding Sites of HIV-1 RT and HIV-2 RT

Figure 4 shows a comparison of the amino terminal sequences of the RTs of HIV-1 strain BH10 and HIV-2 strain ROD, which are the strains used in this analysis. Figure 5 shows a comparison of the putative ATP binding sites of HIV-1 RT and HIV-2 RT. There are a number of differences in the structures. Prominent differences are seen in the portions of the large subunit that form the putative ATP binding pocket. These include: 1) the amino terminus of the RT; 2) the loop that includes amino acids 115–118; 3) the segment that contains amino acids 214 and 215; 4) the region around amino acid 219; and 5) the segment that includes amino acids 41–46.

**Figure 4:**
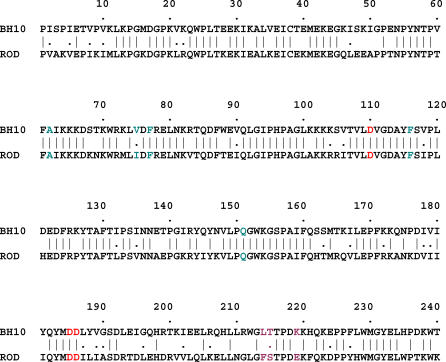
Comparison of the First 240 Amino Acids of HIV-1 RT (Strain BH10) and HIV-2 RT (Strain ROD) The triad of aspartic acid residues critical for polymerase activity is shown in red. The amino acid residues that are altered as part of the Q151M complex in HIV-1 RT are shown in blue; residues 214, 215, and 219 are shown in plum.

**Figure 5:**
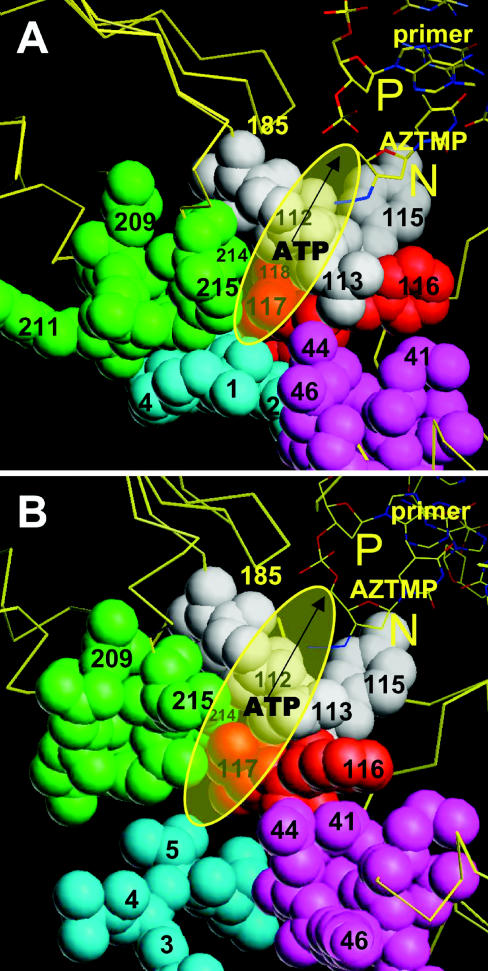
Polymerase Active Site Regions of HIV-1 RT and of HIV-2 RT (A) The HIV-1 RT complex with an AZTMP-terminated primer trapped at the N site (PDB code 1N6Q). (B) Nucleic acid from (A) superposed on the structure of unliganded HIV-2 RT (PBD code 1MU2). The side chains of amino acid residues proximal to the azido group of AZTMP are shown as white spheres, which represent the Van der Waals volumes. The side chains of amino acid residues at the interface of several secondary structure elements of RT are shown in different colors: 209–215 of the large subunit (green spheres), 41–46 of the large subunit (magenta spheres), 116–117 of the large subunit (red spheres), and the N-terminus of the large subunit (cyan spheres). The proposed ATP-binding regions are shown as semitransparent yellow ellipsoids. The direction of the nucleophilic attack of ATP on the AZTMP-terminated primer is indicated by a black arrow.

### The Amino Terminus

There are a number of differences in the first 10 amino acids of HIV-1 RT and HIV-2 RT (Figure 4). Amino acids 1 and 6 are conserved and 2, 5, 7, and 10 are either V or I; the other amino acids are quite different in the two RTs. The amino terminus of the p66 subunit of HIV-1 RT forms the lower part of the putative ATP binding pocket, and helps to organize the segments on the left and right sides of the pocket (Figures 5 and 6). In the vast majority of HIV-1 RT structures, the amino terminus of p66 is tightly associated with the rest of the protein, which helps to define and stabilize the ATP binding pocket. The interaction between the amino terminus of p66 and the rest of HIV-1 RT involves a hydrogen bond between residue 3 of the amino terminus and residue 117 of the fingers subdomain, and additional interactions between residues 2 (and sometimes residue 3) of the amino terminus and residue 213 of the palm subdomain. These interactions vary slightly among the different HIV-1 RT structures; we compared the structures of unliganded HIV-1 RT, complexes with NNRTIs, and complexes with nucleic acid substrates. In the structure of unliganded HIV-2 RT, there is much less contact between the amino terminus of the large subunit and the body of the protein, and there are no corresponding hydrogen bonds [20]. It should be pointed out that neither the HIV-1 RT nor the HIV-2 RT used to generate the crystal structures shown in Figure 6 have the same amino acids at the amino terminus that are present in the corresponding virally produced enzymes. Both recombinant RTs have the normal amino terminus extended by two amino acids (MV in both cases). However, in neither case are the added MV residues visible in the crystal structures. Instead, the first visible amino acid in the large subunit of the majority of the HIV-1 RT crystal structures corresponds to amino acid 1 of virally produced HIV-1 RT. There are structures (including 1FK9, 1COT, and 1EP4) in which amino acid 4 of virally produced RT is the first visible residue at the N terminus. The first visible residue at the N terminus of the large subunit of HIV-2 RT is virally encoded residue 3. In both HIV-1 and HIV-2 RTs the predicted position of the two engineered residues (MV) is not likely to directly affect the formation of the ATP binding pocket [1]. This suggests that the two additional amino acids (MV), which were introduced to facilitate the expression of the HIV-1 RT and HIV-2 RT proteins in *Escherichia coli,* do not markedly affect either structure.

**Figure 6:**
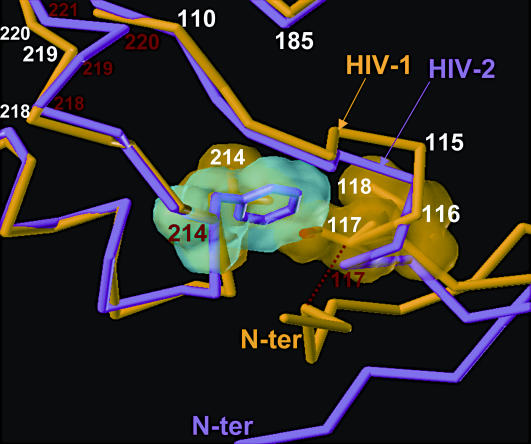
A Comparison of the Structure of an HIV-1 RT/DNA Complex with the Structure of Unliganded HIV-2 RT In the HIV-1 RT complex (PDB code 1N6Q), shown in yellow, the 3′ end of the primer is in the N site, unliganded HIV-2 RT (PDB code 1MU2) is shown in magenta. The residues used for the superposition were 107′112 and 155′215. The Van der Waal radii for the atoms in the amino acid residues at positions 117 and 214 are shown as spheres: yellow for HIV-1 RT and blue for HIV-2 RT. The superposition highlights two major differences between the two enzymes: a) the interaction between the N terminus and residue Ser117 of p66 in HIV-1 RT (red dotted line) is absent in HIV-2 RT because in HIV-2 RT the N terminus has moved away from residue 117 (cyan dotted line); b) in HIV-1 RT, Ser117 interacts with Leu214 1 RT in a way that differs from the interaction of Ser117 and the bulkier Phe214 in HIV-2 RT.

### The Loop That Includes 115–118

The segment that includes amino acids 110–120 is quite conserved in HIV-1 RT and HIV-2 RT; there is only one modest change, at position 118. In HIV-1 RT, 118 is V; in HIV-2 RT, 118 is I (Figure 4). The loop that includes 115–118 is displaced in the larger subunit of HIV-2 RT relative to its position in HIV-1 RT; the loop lies farther from D185 in HIV-2 RT than in HIV-1 RT (see Figure 6). Not only is there a displacement of the amino acid backbone, but there are also substantial differences in the positions of the amino acid side chains of 113, 116, and 117. This may result, in part, from the fact (already mentioned) that there is, in HIV-1 RT, a hydrogen bond between amino acids 3 and 117; this hydrogen bond does not exist in HIV-2 RT. In HIV-1 RT the side chain of 113 lies in front of 116 and 117 and lies somewhat between them. In HIV-2 RT 113 lies above the side chains of 116 and 117, which are much closer to each other than in HIV-1 RT. In HIV-2 RT, it appears that these differences, in particular the positions of the side chains of 113 and 116, would make the putative ATP binding pocket shallower and less able to bind ATP appropriately for the excision reaction.

### Segment That Includes 214 and 215

The segment from 213 to 218 is moderately well conserved when HIV-1 RT is compared to HIV-2 RT (Figure 4). In HIV-2 RT amino acid 214 is an F. In some, but not all, B subtype HIV-1 strains (including BH10, which was used to make the recombinant RTs from which the structures were derived), amino acid 214 is an L. In many other HIV-1 subtypes the predominant amino acid at 214 is an F. HIV-1 RT has a T at position 215; HIV-2 RT an S (Figure 4). In the development of an excision-proficient HIV-1 RT, T215F/Y is a key mutation. For the HIV-1 isolate BH10 used in these experiments, the codon for T215 is ACC. Mutation to either phenylalanine (TTC) or tyrosine (TAC) would require two nucleotide substitutions. In contrast, the codon for S215 in HIV-2 RT can be TCT (e.g., strains ROD and D194) or TCC (e.g., strain SBLISY). A single nucleotide change is sufficient to convert this codon to F (TTT or TTC) or Y (TAT or TAC). Even though it is simpler to change the amino acid at 215 to an F or Y in HIV-2 than in HIV-1 (see below), this mutation does not appear to be the favored pathway for the development of AZT resistance in HIV-2. The aromatic residue (either F or Y) at 215 in the p66 subunit of HIV-1 RT is thought to interact with the adenine ring of ATP. This suggests that the position of the amino acid side chain at position 215 (relative to the polymerase active site) would be crucial for ATP binding and excision. In both HIV-1 and HIV-2 RT, the position of the 215 side chain appears to be affected by the interaction between residues 214 and 118. In HIV-1 RT, V118 and L214 make a hydrophobic interaction. In HIV-2 RT, the corresponding residues are larger (F214 and I118) and the interaction results in repositioning of the corresponding segments. As a result, the side chain of S215 is closer to the active site of HIV-2 RT than is the corresponding T215 of HIV-1 RT, and the position of the S215 of HIV-2 RT is stabilized by a hydrogen bonding interaction with the main chain at position 112 (Figure 6). The larger F at 214 of HIV-2 RT may not only affect the position of the side chain of 215 but also contribute to the shallowness of the putative ATP binding pocket in HIV-2 RT (and potentially in those HIV-1 isolates that have an F at this position). In comparing the ability of wild-type HIV-1 RT and HIV-2 RT to carry out ATP-mediated AZTMP excision, it is worth noting that the threonine present at position 215 in HIV-1 RT is modestly more hydrophobic than is the serine found at 215 in HIV-2 RT. Because of the importance of the T215F/Y mutation, even this small difference in hydrophobicity, coupled with the more favorable position of the T215 side chain, would enhance the ability of wild-type HIV-1 RT to bind ATP appropriately for excision relative to wild-type HIV-2 RT.

### The Region around 219

The amino acids from 216–220 are conserved, except for 219, which is K in HIV-1 RT and E in HIV-2 RT (Figure 4). Despite this similarity, there is, in the larger subunit of HIV-2 RT, a transposition of the amino acid backbone of HIV-2 RT relative to HIV-1 RT so that amino acid 219 of HIV-2 RT is much closer to D185 than it is in HIV-1 RT (Figure 7). This shift also causes the approximate alignment of K219 in HIV-1 RT with K220 of HIV-2 RT (Figure 7). It is not clear whether this difference would affect ATP-mediated excision, and, if it does, whether the effect of moving E219/K220 in HIV-2 RT relative to the active site would be favorable or unfavorable for excision. This difference in the region around 219 may be connected to changes seen in the loop that carries the active site amino acids 185 and 186 (Figure 7). The only available HIV-2 RT structure is unliganded (there is, in the structure, no bound inhibitor, nucleic acid, or incoming dNTP) [19,20]. In HIV-1 RT, a comparison of the available structures, unliganded and liganded, shows that binding an incoming dNTP appears to cause the 185–186 loop (β9–β10) to move “down” relative to the position of the nucleic acid (Figure 7). The binding of tenofovir diphosphate at the polymerase active site moves the 185/186 loop down and also brings the side chain of 219 down toward 185/186 (Figure 7), relative to the position the 219 side chain occupies when a normal dNTP is bound [21]. Despite the fact that the HIV-2 RT structure was generated with an unliganded protein, the 185/186 loop is in a “down” position relative to unliganded HIV-1 RT. As has already been pointed out, the backbone position of K219 in HIV-1 RT is approximately the same as the backbone position of K220 in HIV-2 RT. These changes could affect ATP-mediated excision reaction. If these specific interactions affect the ability of HIV-2 RT to bind ATP in a favorable way, the interactions would have to involve portions of ATP other than the beta and gamma phosphates because any unfavorable interactions at these positions would be expected to interfere with the polymerization reaction. In the polymerization reaction, the gamma and beta phosphates of the incoming dNTP are expected to interact with RT at positions that are very similar to the positions where the beta and gamma phosphates of ATP are believed to bind in the excision reaction. As has already been discussed, HIV-2 RT is a fully competent polymerase and carries out pyrophosphate-mediated excision with an efficiency similar to HIV-1 RT.

**Figure 7:**
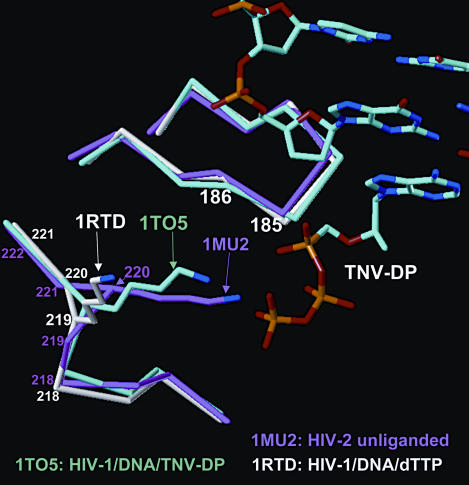
Position of the Side Chain of Amino Acid Residue K219 in HIV-1 RT and K220 in HIV-2 RT Unliganded HIV-2 RT (shown in magenta; PDB code 1MU2) and HIV-1 RT/DNA/dTTP (shown in white with the dTTP and DNA omitted for clarity; PDB code 1RTD), is superimposed on the Cα protein backbone of HIV-1 RT/DNA/tenofovir-diphosphate (shown in cyan; PDB code 1TO5). The residues used for the superposition were 107–112 and 155–215. The alignment shows that K220 of HIV-2 RT is the residue structurally equivalent to K219 in HIV-1 RT.

### The Segment That Includes 41–46

Amino acids 41–45 are conserved between HIV-1 RT and HIV-2 RT; however, amino acids 46–50 are not (Figure 4). Residue 46 is K in HIV-1 RT; it is Q in HIV-2 RT. When the two structures are compared, the relative arrangements of the amino acid side chains in the larger subunit at positions 41, 44, and 46 are quite different. In HIV-1 RT, 44 and 46 lie close together near the lower end of the ATP binding pocket. In HIV-2 RT, 41 lies next to 44, but 46 is displaced well away both from 44 and from the putative ATP binding pocket. These differences appear to be connected to the changes in the amino terminus, which lies close to 44 and 46. There are good reasons to think that both 41 and 44 (as well as 118) may play a role in positioning ATP for excision in HIV-1 RT. M41L is one of the canonical mutations involved in AZT resistance in HIV-1 RT; this suggests that amino acid 41 plays a direct or indirect role in ATP binding. HIV-1 RT carrying the canonical mutations associated with AZT resistance (M41L, D67N, K70R, T215F/Y, K219Q) excises AZTMP efficiently, but cannot excise 3TCMP to any significant extent. The addition of E44D and V118I confers 3TC resistance in the absence of the M184V mutation, apparently by enhancing 3TCMP excision, suggesting that these mutations affect the positioning of ATP and, as a result, the efficiency of 3TCMP excision [1].

### A Comparison of the Regions around Q151 in HIV-1 RT and HIV-2 RT

In the sections presented above, the differences between the activity and structure of HIV-1 RT and HIV-2 RT were considered in light of their effects on the putative ATP binding site. If, as we propose, wild-type HIV-2 RT binds ATP less efficiently than wild-type HIV-1 RT, this would explain why HIV-2 RT does not prefer to use ATP-dependent excision for AZT resistance. As described above, AZT resistance in HIV-2 appears to involve the Q151M mutation, which has been shown to affect NRTI incorporation in HIV-1 RT [1]. This mutation is occasionally selected in HIV-1 RT in response to NRTI combination therapy; however, it has only been observed in approximately 5% of treated patients [1]. In contrast, in HIV-2 infected patients receiving NRTI-containing therapies, Q151M has been reported to be present in more than 45% of the patients examined [11], and other groups have also reported a high frequency of the Q151M mutation in HIV-2 patients receiving NRTI therapy [13–15]. It is possible that HIV-2 RT prefers to use the Q151M pathway for AZT resistance, at least in part, because wild-type HIV-2 RT incorporates AZTTP less efficiently, and is already partway along a pathway leading to reduced AZTTP incorporation. It is also possible that the Q151M mutation is more favorable in the context of HIV-2 RT than HIV-1 RT. As discussed above, the Q151M mutation causes an increase in resistance to AZTTP in both HIV-1 RT and HIV-2 RT (Figure 2). However, in part because the wild-type HIV-2 RT is already more resistant to AZTTP than is the HIV-1 variant Q151M, the presence of Q151M makes the HIV-2 RT highly resistant to AZTTP (Figure 2). Position 151 is, in the larger subunit, part of the dNTP binding site and we compared this region in the two RTs (Figure 8). Because Q151 is in a region of the large subunit that interacts with both the nucleic acid and the incoming dNTP, we compared the structure of unliganded HIV-2 RT to both an unliganded HIV-1 RT structure and to an HIV-1 RT structure that contains both a bound DNA and an incoming dNTP. We considered the position of 151 itself and the segment that contains 151. We reexamined the segment that carries 113–118, which had been considered (and discussed) as part of the analysis of the putative ATP binding site, to investigate its role in the binding and incorporation of an NRTITP. We also looked at the positions where secondary mutations arise in HIV-1 RTs that acquire the Q151M mutation (A62V, V75I, F77L, and F116Y).

**Figure 8:**
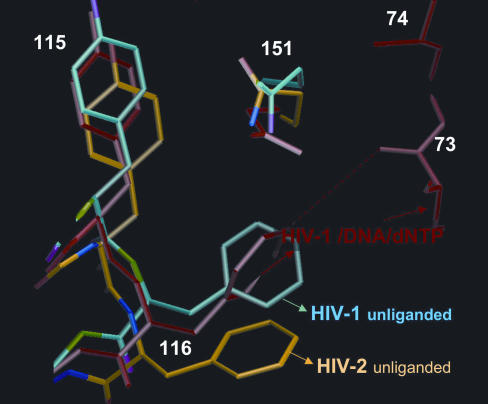
Superposition of HIV-1 RT and HIV-2 RT Showing the Possible Effects of a Phe116Tyr Mutation Unliganded HIV-2 RT (PDB code 1MU2) is shown in yellow, unliganded HIV-1RT (PDB code 1DLO) is shown in cyan, and HIV-1 RT/DNA/dTTP (PDB code 1RTD) is shown in red. The superposition shows that a Phe116Tyr mutation in HIV-1 RT would affect the interactions of position 116 with the main-chain carbonyl of Lys73 in the fingers subdomain of HIV-1 RT (dotted line). However, the same mutation in HIV-2 RT is not likely to cause similar interactions of Tyr116 with the fingers subdomain of HIV-2 because the differences in the position of the small helix that carries residues 115–117 in HIV-2 RT make the interaction unlikely.

### The Segment That Includes Q151

The segment from148 to 161 is conserved between HIV-1 RT and HIV-2 RT (Figure 4); in addition, the Q151 residue is in a very similar position in the two RTs. In the ternary complex of HIV-1 RT, Q151 is near R72, L74, Y115, and F116 (Figure 8). Q151 also interacts with the main chain at position 73. These residues form part of the dNTP binding pocket, specifically that portion of the pocket that underlies the deoxyribose ring. Q151 interacts with the 3′-OH of a normal dNTP and, through R72, with the phosphates. Because a 2′,3′-dideoxynucleotide triphosphate (ddNTP) lacks the 3′-OH that Q151 would normally interact with, the network of hydrogen bonds differs in a complex with a ddNTP and Q151 interacts only with R72 [1]. The substitution of a methionine for the glutamine normally present at 151 enhances the ability of the enzyme to discriminate against the incorporation of nucleoside analogs that lack a 3′-OH [1,8–10]. Although we do not have a ternary structure of HIV-2 RT, it is likely, because the Q151 residue and its immediate environment are similar in the two RTs, and because the resistance mutations are the same (Q151M), that the underlying resistance mechanism is the same. In the case of HIV-1, RTs that carry the Q151M mutation are less sensitive to AZTTP and ddNTPs than wild-type RT, and the addition of secondary mutations (A62V, V75I, F77L, and F116Y) causes a further decrease in the sensitivity to these analogs. The fitness cost, if any, of the Q151M mutation in HIV-2 is not known (discussed below).

### The Segment That Includes 113–118

Despite the fact that the sequence of the segment that carries 113–118 is conserved between HIV-1 RT and HIV-2 RT, the exact position of this segment differs slightly when unliganded HIV-1 RT is compared with the HIV-1 RT ternary complex; however, it is displaced considerably farther in the unliganded HIV-2 RT structure, in part because (as has already been discussed) there is a hydrogen bond between amino acid 3 and amino acid 117 in HIV-1 RT which does not exist in HIV-2 RT. We do not yet have a ternary structure of HIV-2 RT. The region that includes 113, 114, and 115, which interact with the phosphates and the deoxyribose of the incoming dNTP, is displaced by 1–1.5 Å in HIV-2 RT (Figures 6 and 8). A comparison of HIV-1 RT and HIV-2 RT suggests that this could alter the interactions of these amino acids with the incoming dNTP and/or the exact position the incoming dNTP has in the HIV-2 RT ternary complex. Alternatively, it is possible that the binding of the incoming dNTP causes a greater movement of this segment of HIV-2 RT than HIV-1 RT, and the positions these amino acids (and the incoming dNTP) occupy in the HIV-2 RT ternary complex are more similar to their positions in the HIV-1 RT ternary complex than the positions of the amino acid side chains in the 113–116 segment of the unliganded HIV-2 RT would suggest.

### The Mutations That Accompany Q151M in HIV-1 RT

There is a family of mutations (A62V, V75I, F77L, and F116Y) that are often found with Q151M in HIV-1 RT. The data available in the Stanford database suggest that this suite of mutations does not accompany the Q151M mutation in HIV-2 RT in the same way these mutations accompany the Q151M mutation in HIV-1 RT. In part this may be due to the fact that there are much fewer data for AZT-resistant HIV-2 RTs. However, the structures suggest that at least some of these secondary mutations might not have the same importance in HIV-2 RT as in HIV-1 RT. In wild-type HIV-2 RT, the amino acid usually present at position 75 is already an isoleucine (it is possible that the presence of I75 contributes to the low level of AZTTP incorporation seen with wild-type HIV-2 RT). In HIV-1 RT, F77 interacts directly with V75, and it may be that the reason the F77L change is found in HIV-1 RT is to help compensate for the V75I mutation. Because HIV-2 RT already has I75, the F77L change may not be needed. The A62V mutation does not appear to have a major effect either in the structure of HIV-2 RT or in the behavior of HIV-1 RT. Neither position 62 nor 77 have any direct interactions with the nucleic acid or the incoming dNTP, nor are either of these positions in direct contact with Q151. In contrast, it appears that the F116Y is a more important secondary mutation. In wild-type HIV-1 RT, Q151M interacts with R72. This interaction is important in the two different complexes that HIV-1 RT forms with dNTPs and ddNTPs. Substitution of a methionine for the glutamine normally present at position 151 will alter the interaction of R72 and Q151. The substitution of a tyrosine for the phenylalanine normally found at position 116 in HIV-1 RT allows the formation of a hydrogen bond between the OH of the tyrosine and the main chain at position 73. This may help compensate for the altered interaction between the methionine at 151 and R72 in the Q151M mutant and help to stabilize the dNTP binding pocket in the mutant HIV-1 RT. In contrast, the position of the phenylalanine side chain of F116 in unliganded HIV-2 RT is sufficiently different that, unless there is a significant rearrangement of this region when an incoming dNTP binds, the F116Y mutation would not have the same effect in HIV-2 RT that it has in HIV-1 RT. In unliganded HIV-2 RT the side chain of 116 is so far away from the main chain at position 73 that introducing a tyrosine at position 116 would not create the same hydrogen bond that can be made in the F116Y mutant of HIV-1 RT, unless there is significant repositioning of the segment that carries this residue when substrate is bound (Figure 8).

## Discussion

The ability of HIV-1 RT to develop resistance to AZT by the excision pathway poses serious problems for therapy with this NRTI. The fact that AZT-resistant HIV-1 RTs can acquire additional mutations, for example the insertions in the fingers subdomain that allow them to excise a broad range of NRTIs, poses a challenge for this entire class of drugs. Despite the fact that the full development of high-level AZT resistance via the excision pathway requires multiple mutations, and the fact that the key mutation, T215Y/F, requires two nucleotide changes, excision pathway is the commonly used pathway for development of AZT resistance in HIV-1. What makes this somewhat surprising is that the virus has an alternative: the Q151M mutation confers resistance to AZT and arises in patients given certain NRTI combinations (particularly AZT in combination with ddI and ddC) [1,8–10]. Like T215Y/F, the mutation Q151M also requires two nucleotide changes and requires additional mutations to increase the level of resistance to various NRTIs. What makes HIV-1′s use of the excision pathway somewhat more puzzling is this: the related virus, HIV-2, does not appear to use the excision pathway as frequently when challenged with AZT. Moreover, as has already been discussed, changing S215 to either tyrosine or phenylalanine requires only a single nucleotide change, and thus should arise more readily in HIV-2 RT than in HIV-1 RT. Although the number of patients is much smaller, and so the data are not as robust, it appears that in HIV-2–resistance to AZT usually involves the Q151M mutation, and it has been proposed that the two viruses use different pathways to develop AZT resistance [11–16].

We compared HIV-1 RT and HIV-2 RT both structurally and biochemically. The biochemical analysis suggests that wild-type HIV-1 RT is more susceptible than HIV-2 RT in terms of incorporating AZTTP, but that HIV-1 RT has a better nascent ATP binding site than HIV-2 RT. The structural comparisons show that there are a number of differences in the putative ATP binding site that can account for the difference in the ability of these two RTs to bind ATP. In HIV-2 RT, the putative ATP binding pocket appears to be shallower and less well defined. This suggests that one of the reasons HIV-1 chooses the excision pathway for AZT resistance is because the wild-type enzyme already has a nascent ATP binding site that is sufficiently good to allow the wild-type enzyme to carry out moderately efficient ATP-mediated excision. As a consequence, it is relatively simple for the virus to mutate in a way that enhances the ability of HIV-1 RT to bind ATP appropriately and to carry out ATP-mediated excision efficiently. In contrast, because HIV-2 RT does not have a particularly good nascent ATP binding site, it is much more difficult for mutations to create an effective ATP binding site. This makes the alternative pathway (AZT resistance via the Q151M mutation) the more attractive alternative for HIV-2, particularly because HIV-2 RT is, relative to HIV-1 RT, less susceptible to incorporating AZTTP.

We found that HIV-1 RT has a lower K_m_ and a higher k_cat_ for AZTTP than HIV-2 RT. However, HIV-1 RT also has a lower K_m_ and a higher k_cat_ for dTTP than HIV-2 RT. Our data were derived from the analysis of the incorporation of either dTTP or AZTTP in a single cycle of incorporation assay; this means the data can be compared directly. We suggest that the fact that AZT is poorly phosphorylated within the cell could make the difference in K_m_ for AZTTP (0.17 μM for HIV-1 RT versus 0.75 μM for HIV-2 RT) an important difference because low levels of AZTTP within a cell would make the K_m_ for AZTTP a limiting factor. However, there is a significant problem in using this type of single-cycle kinetic assay to try to understand the resistance or susceptibility of HIV-1 or HIV-2 viruses to inhibition by AZT. The relative efficiency of incorporating AZTTP and dTTP is quite sequence-dependent [22], as is the excision reaction [23,24]. This means that kinetic assays done using any specific sequence give data that may not reflect the ability of the RT to incorporate (or excise) a particular nucleoside analog (like AZT) and that assays that measure incorporation (and excision) over several different sites give an average value that more accurately reflects what happens when RT copies the viral genome. Thus, the data in Figure 2 better reflects the overall ability of HIV-1 RT and HIV-2 RT to differentiate AZTTP and dTTP than do the kinetic data in Table 1.

In considering why HIV-1 RT prefers to use the excision pathway and HIV-2 RT the Q151M exclusion pathway, we also compared the region around Q151, and the positions where there are secondary mutations that often accompany Q151M in HIV-1 RT. The region around Q151 is involved in binding the incoming dNTP and it is not surprising that this region is (in terms of amino acid sequence) quite conserved in the two RTs. However, when the unliganded structures are compared, there are obvious differences in the positions of the amino acids on the 113–118 segment. We do not yet have a ternary structure of HIV-2 RT with a bound incoming dNTP, so it is difficult to know whether there are significant differences in the interactions of HIV-2 RT and the incoming dNTP. One of the secondary mutations seen in HIV-1 RT, V75I, is present in wild-type HIV-2 RT; moreover the secondary mutation that appears to be the most significant in HIV-1 RT, F116Y, would not be expected, based on the available unliganded structure, to have the same effect in HIV-2 RT that it has in HIV-1 RT. High-level resistance to ddNTPs and AZTTP in HIV-1 RT requires not only the Q151M mutation, but other secondary mutations. Based on our in vitro data, it appears that in HIV-2 RT, the Q151M mutation, along with the naturally occurring I75 residue, is sufficient to generate high-level resistance to AZTTP. This is supported by the fact that wild-type HIV-2 RT is already more resistant to AZTTP than is wild-type HIV-1 RT or the HIV-1 RT variant Q151M (Figure 2). This difference has also been seen when the AZT susceptibility of HIV-1 and HIV-2 viral replication were compared [16]. If only one amino acid change is required to generate high-level AZT resistance in HIV-2 RT via the Q151M exclusion pathway, this would favor the Q151M exclusion pathway in HIV-2 RT, while the fact that multiple amino acid substitutions are needed to give high-level AZT resistance in HIV-1 RT would mean that the Q151M pathway would be less favored.

Another consideration is the fact that the Q151M mutation requires two nucleotide changes (CAG to ATG). While it is possible that the two changes could occur simultaneously, it is more likely that the changes occur one base at a time. This would require either a Q151L (CAG to CTG) or a Q151K (CAG to AAG) intermediate. There may be differences in the ability of the HIV-1 RT and HIV-2 RT to tolerate the intermediate mutations that are needed to get to the final Q151M mutation. There are data that suggest that both of these intermediates are quite deleterious for the activity of HIV-1 RT, which could make it difficult for HIV-1 to generate the Q151M mutation [25–27]. There are no published data on the effects of the intermediate mutations at Q151 on HIV-2 RT activity; however, the Q151L mutant has been detected in HIV-2–infected patients, suggesting that at least this mutant may be less deleterious for HIV-2 RT than for HIV-1 RT [11,14].

As has already been suggested, one way to look at the problem is that HIV-1 RT, which readily incorporates AZTTP, also has a relatively good nascent ATP binding site and can be thought of as being part way along the pathway to developing AZT resistance via excision. In contrast, wild-type HIV-2 RT incorporates AZTTP less well and has a less well-developed ATP binding site, so it can be considered to be already on a pathway of becoming resistant by decreasing its incorporation of AZTTP. Viewed in this light, the development and exploitation of the excision pathway by HIV-1 RT is, from the point of view of those trying to develop more effective therapies, a very unfortunate coincidence. There is no reason to think that the nascent ATP binding site that HIV-1 RT exploits in the development of AZT resistance has any important role in RT or reverse transcription in the absence of AZT. This idea is reinforced by the data showing that HIV-2 RT has a much less well-developed nascent ATP binding site: if the ability to bind ATP for the purpose of AZTMP excision was connected to some important underlying function, or had an important role in reverse transcription, then this ATP binding site would likely be found in HIV-2 RT, too. These observations also reinforce the opportunistic nature of the evolutionary events that lead to drug resistance in HIV-1 and HIV-2: modest differences in the ability of the two wild-type enzymes to incorporate AZTTP and to bind ATP and excise AZTMP cause them to prefer different paths when they develop resistance to AZT, an idea also proposed for the different NRTI resistance mutations seen in HIV-1 RT [28].

## Materials and Methods

### Preparation of HIV-1 and HIV-2 RTs.

The open reading frame encoding wild-type HIV-1 RT (strain BH10) was cloned into a plasmid-containing HIV-1 PR open reading frame as previously described [5]. A similar plasmid encoding the open reading frame for HIV-2 RT (strain ROD) and the HIV-2 PR was the generous gift of Dr. Amnon Hizi (Tel Aviv University) and has been previously described [17,18]. The plasmids are based on the expression vector pT5m, and were introduced into the E. coli strain BL21 (DE3) pLysE. After induction with isopropyl β-D-thiogalactopyranoside, the plasmids express both the p66 forms of HIV-1 RT and HIV-1 PR, or the p68 form of HIV-2 RT and HIV-2 PR. Approximately 50% of the over-expressed p66 HIV-1 RT or p68 HIV-2 RT is converted to the p51 or p54 form by the co-expressed PR. The heterodimers accumulate in E. coli and were purified by metal chelate chromatography [5].

### Polymerase assays.

The polymerase assays were done as previously described [5]. For each sample, 0.25 μg of single-stranded M13mp18 DNA (New England Biolabs, Beverly, Massachusetts, United States) was hybridized to 0.5 μl of 1.0 OD/ml of the −47 sequencing primer (New England Biolabs). The template/primer (T/P) was suspended in 100.0 μl of 25 mM Tris (pH 8.0), 75 mM KCl, 8.0 mM MgCl_2_, 100.0 μg/ml BSA, 10.0 mM CHAPS, 2.0 mM DTT, 10.0 μM each of dATP, dTTP, dCTP, and dGTP, and 2.0 μM [α−^32^P]dCTP. Extension was initiated by the addition of 1.0 μg of wild-type RT. The mixture was incubated for 30 min at 37 °C, then the reaction was halted by the addition of 3 ml ice-cold trichloroacetic acid. Precipitated DNA was collected by suction filtration through Whatman GF/C glass filters. The amount of incorporated radioactivity was determined by liquid scintillation counting. Inhibition of polymerization by AZTTP was done in a similar manner, except that various concentrations of AZTTP were added to the reaction. The activity of the enzyme was considered to be 100% in the absence of the AZTTP; the decreased level of incorporated radioactivity in the presence of AZTTP was normalized to this value.

### Processivity assay.

The processivity assay has been previously described [5]. In brief, for each sample to be assayed, 0.5 μl of 1.0 OD/ml −47 sequencing primer (New England Biolabs) was 5′ end-labeled with [γ−^32^P]ATP and T4 polynucleotide kinase. After purification, the labeled primer was annealed to single-stranded M13mp18 DNA (1.0 μl of a 0.25 μg/μl DNA stock for each sample to be assayed) by heating and slow cooling. The labeled T/P was resuspended in RT buffer, which is 25 mM Tris (pH 8.0), 75 mM KCl, 8.0 mM MgCl_2_, 100 μg/ml BSA, 10.0 mM CHAPS, and 2.0 mM DTT. One microgram of wild-type HIV-1 or HIV-2 RT was added to each tube and allowed to bind to the labeled template primer for 2 min. Extension was initiated by the addition of dNTPs, to a final concentration of 10.0 μM each, plus 0.5 U/ml poly(rC)•oligo(dG), which prevents RT from rebinding to the labeled primer by binding the RT after it disassociates from the labeled T/P.

### Pyrophosphorolysis.

ATP- and NaPPi-dependent pyrophosphorolysis analysis was done as previously described [5]. A synthetic DNA oligonucleotide (5′-GTCACTGTTCGAGCACCA-3′) (Biosource, Camarillo, California, United States) was 5′ end-labeled, purified, then annealed to the template (5′-AATCAGTGTAGACAATCCCTAGCAATGGTGCTCGAACAGTGAC-3′). The 3′ end of the primer was then blocked by the addition of AZTMP. After purification of the blocked T/P, the T/P was incubated in RT buffer with HIV-1 RT or HIV-2 RT in the presence of various concentrations (described in the figure legends) of either NaPPi or ATP for 15 min. The reactions were halted by the addition of EDTA; the salts and nucleotides were removed by passage through a CentriSep column (Princeton Separations, Adelphia, New Jersey, United States) and the T/P precipitated by the addition of isopropyl alcohol. The products were fractionated on a 15% polyacrylamide sequencing gel. The total amount of T/P (blocked and unextended plus deblocked and extended) and the amount of full-length product (deblocked and extended to the end of the template) were determined using a Phosphorimager.

### Single dNTP incorporation kinetics.

A synthetic oligonucleotide primer was 5′ end-labeled, purified, then annealed to the template as described above. The labeled T/P was resuspended in polymerase buffer (final concentration of 15.0 nM T/P, 20.0 mM Tris [pH 8.0], 75.0 mM KCl, 16.0 mM MgCl_2_, 2.0 mM DTT, 0.1 mg/ml acetylated BSA, 10.0 mM CHAPS, 3.0 nM RT). The reactions were equilibrated at 37 °C for 5 min, then the reactions initiated by the addition of dTTP or AZTTP. Final concentrations of the dNTPs ranged from 0.4 μM to 200.0 μM. The reactions were allowed to proceed for 1.0 min, and the reactions halted by the addition of EDTA; the salts and nucleotides were removed by passage through a CentriSep column (Princeton Separations) and the T/P precipitated by the addition of isopropyl alcohol. The products were fractionated on a 15% polyacrylamide sequencing gel. The product of the reaction is the amount of primer extended by one nucleotide; the concentration of the product was determined using a Phosphorimager. The concentration of substrate [S] is the concentration of dNTP in the reaction, while velocity (v) is the amount of product (nM of primer + 1) generated in 1 min at the given [S]. The data was plotted using the Woolf-Augustinsson-Hofstee plot: v versus v/[S]. The line of best fit was determined by linear regression and the V_max_ and K_m_ were determined from the intercepts of the plot.

## Abbreviations

ddNTP: 2′,3′-dideoxynucleotide triphosphate
dNTP: 3′-deoxynucleotide triphosphate
HIV: human immunodeficiency virus
N site: nucleotide binding site
NNRTI: nonnucleoside reverse transcriptase inhibitor
NRTI: nucleoside analog reverse transcriptase inhibitor
NRTIMP: nucleoside analog reverse transcriptase inhibitor monophosphate
NRTITP: nucleoside analog reverse transcriptase inhibitor triphosphate
RT: reverse transcriptase
